# Supplementary material for: The Impact of Vp-Porin, an Outer Membrane Protein, on the Biological Characteristics and Virulence of Vibrio Parahaemolyticus
Source: Biology (Basel). 2024 Jun 28;13(7):485. doi: 10.3390/biology13070485 (PMC11273978; doi:10.3390/biology13070485)
Supplement: Supplementary file 1 [file biology-13-00485-s001.zip › biology-3067577-supplementary.pdf]

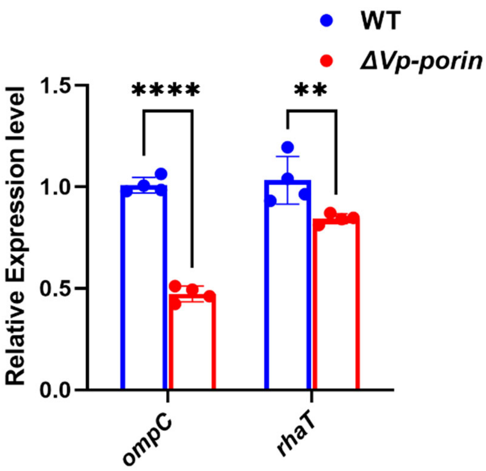

Figure S1 The effect of the deletion of *Vp-porin* on the expression of genes adjacent to *Vp-porin*. qRT-PCR analysis of the transcription levels of ompC and rhaT between WT and Δ*Vp-porin*. The data are presented as the mean ± SD (n = 4). *$p < 0.05$; **$p < 0.01$; ***$p < 0.001$.
